# Hepatitis E prevalence in French Polynesian blood donors

**DOI:** 10.1371/journal.pone.0208934

**Published:** 2018-12-07

**Authors:** Chloé Dimeglio, Frédéric Beau, Julien Broult, Patrice Gouy, Jacques Izopet, Stéphane Lastère, Florence Abravanel

**Affiliations:** 1 CHU Toulouse, Hôpital Purpan, *Laboratoire de virologie*, Centre national de référence du virus de l’hépatite E, Toulouse, France; 2 Centre hospitalier de Polynésie française, Centre de transfusion sanguine, Pirae, Polynésie française; 3 INSERM UMR 1043; CNRS UMR 5282, Centre de Physiopathologie de Toulouse Purpan (CPTP), Toulouse, France; 4 Centre hospitalier de Polynésie française, Laboratoire de biologie médicale, Pirae, Polynésie française; University of Cape Town, SOUTH AFRICA

## Abstract

The HEV seroprevalence in mainland France is elevated (22.4%). In contrast, anti-HEV seroprevalence appears to be lower in Oceania. However, none is available for French Polynesia. We assessed the anti-HEV IgG and IgM prevalence on samples from 300 consecutive blood donors living on Tahiti and Moorea islands. Epidemiological information was collected using a specific questionnaire. Overall IgM seroprevalence was 0.6% and overall IgG seroprevalence was 7.7%. The presence of anti-HEV IgG was associated with increasing age (p = 0.01), eating chicken offal (p = 0.01) and cooked rabbit (p = 0.02). Conversely, eating *fafaru*—traditional Polynesian condiment—was associated with a lower rate of anti-HEV IgG (p<0.01).). All donors who surfed or practiced *va’a* (traditional outrigger canoë) were HEV seronegative. The Polynesian lifestyle and the particular food consumption patterns—especially the very well cooked pork—may be the key to understand the low HEV seroprevalence in French Polynesia.

## Introduction

HEV is now recognized as one of the most common causes of acute viral hepatitis in the world [1]. The burden of HEV in industrialized countries on several continents has been investigated, with studies on the seroprevalence of anti-HEV immunoglobulin G (IgG) and the frequency of HEV RNA among asymptomatic blood donors worldwide. The seroprevalence of anti-HEV IgG in Europe varies from 4.7% in Scotland [2] to 29% in Germany [3]. In contrast, anti-HEV seroprevalence appears to be lower in Oceania, but data are available for only two countries: it is 4–9.7% in New Zealand [4, 5] and 6% in Australia [6]. In France, the overall seroprevalence in mainland France and three overseas territories among blood donors was 22.4% (range 8.0% to 86.4%) with significant geographical difference and hyperendemic areas in the southern part and in the north-east part of France [7]. However, only few French department (3/98) had anti-HEV IgG seroprevalence <10% [7].

We have data on HEV seroprevalence in mainland France and three overseas territories [7] but none are available for French Polynesia, a French overseas collectivity of 275 918 inhabitants, located in the South Pacific Ocean (ISPF 2017 census). The last census asking questions regarding ethnicity indicated that two-thirds of the population was ethnic Polynesians, about 20% were Métis, 10% were Europeans, and 10–15% were East Asian (mainly Chinese) [8].

The first case of acute hepatitis E was discovered in 2014. This not very surprising discovery, and the project to set up plasma production from local donations, led to the implementation of a seroprevalence study to document the exposure to the virus among French Polynesia blood donors.

## Patients and methods

### Study population

We performed a cross sectional study. Anti-HEV IgG and IgM were tested on samples from 300 consecutive blood donors living on Tahiti and Moorea islands (75% of the Polynesian population) between August 25 and September 17, 2014.

### Questionnaire

The procedure followed was based the one usually used by the French Polynesia blood bank: donor information, questionnaire completed by the donor, written consent form for donation and use of data and blood for non-therapeutic purposes, interview and pre-donation medical examination, anonymization of the file and data.

Each participating donor also completed a specific, structured questionnaire to document putative epidemiological factors associated with the presence of anti-HEV (S1 and S2 Files). These factors included the type of dwelling (apartment, house, farm, institution) and its wastewater system (mains sewer, septic tank), professional and extraprofessional activities (gardening, hunting, surfing or *va’a* (Polynesian traditional canoe), and travel history outside Polynesia. We recorded information about the donor’s usual contact with pets and/or domestic farm animals and eating habits. The questions concerned the consumption of meat (animal), meat products (ham, sausages, pâté), fish and shellfish, including *fafaru (Tahitian seasoning)*, and uncooked and unpeeled vegetables. *Fafaru* is a traditional Polynesian condiment prepared by fermenting fish with crushed raw shrimp heads marinated in clean seawater. We also recorded the type of cooking and the things that were eaten uncooked. Last, we recorded the source of drinking water (bottled, tap, untreated private well).

### Laboratory methods

Anti-HEV IgG and anti-HEV IgM antibodies were detected with the Wantai HEV IgG EIA and Wantai HEV IgM EIA kits (Wantai Biologic Pharmacy Enterprise, Beijing, People’s Republic of China) according to the manufacturer’s instructions. The limit of detection of the Wantai IgG anti-HEV assay is 0.25 WHO units/mL [8]. Anti-HEV IgM positive samples were tested for HEV RNA using an accredited in-house assay (ISO15189) [9].

### Statistical analysis

An Excel file was constructed to contain the anonymised questionnaire data, which were then analyzed using Stata version 14 (StataCorp LP, College Station, TX). The parameters codified quantitatively according to the frequencies of consumption in the questionnaire were all recoded as binary: 0 = never and 1 = consumption or exposure at least once (once a week, once a month or once a year). This is also the case for the journey frequencies. All variables were binary variables except the age. No imputation is performed.

We have estimated HEV seroprevalence (IgG and IgM) and its 95% exact binomial confidence interval.

Demographic and lifestyle factors and the consumption variables associated with our outcome, anti-HEV IgG, were evaluated using univariate analyses. Chi-squared and Fisher’s exact tests (if an expected cell frequency is less than 5) for the binary variables, Student’s *t* test with unequal variances was used for the age when the assumption of normality was verified. We used a logistic regression model to identify variables independently associated with anti-HEV IgG; the variables used all had a P value <0.20 after univariate analysis. We included, in addition to the consumption variables, the age and sex of the blood donors to account for some potential cofounders. Statistical significance was set at p<0.05. Possible interactions between independent risk factors were tested by including proper cross-product terms in the logistic models, and likelihood ratio tests comparing models with and without the interaction term were used to estimate the significance of the interaction.

## Results

**Fig 1:**
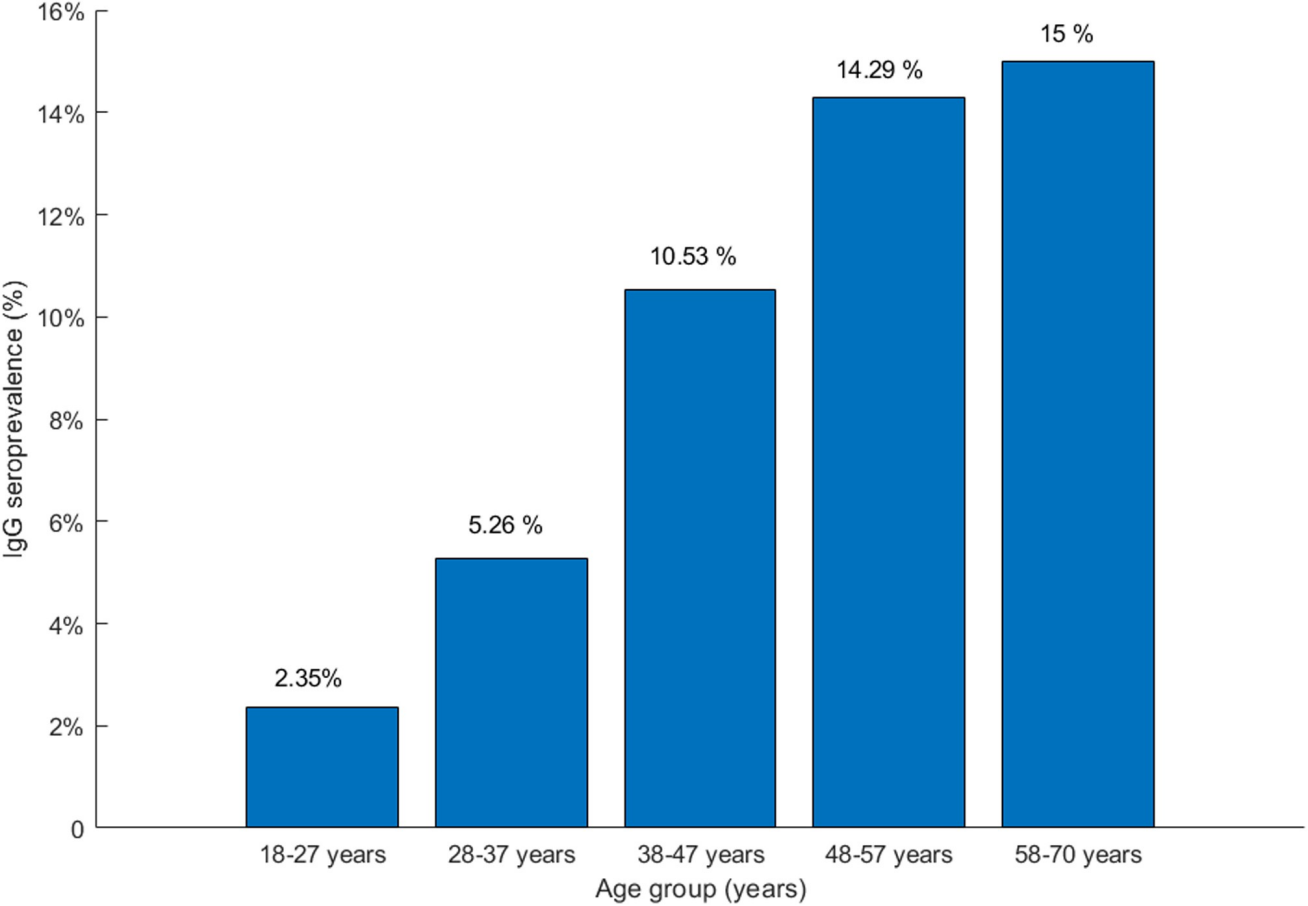
IgG prevalence by age group in French Polynesia.

The 300 blood donors who completed the epidemiological questionnaire included 161 (53.7%) males. The median age was 35 years (interquartile range 26.5–46). Of the 300 blood donors, 23 were anti-HEV IgG positive, and two of these donors were also IgM positive. Overall seroprevalence was 7.7% (95% CI: [4.9% - 11.3%]) for anti-HEV IgG and 0.6% (95% CI: [0.1% - 2.4%]) for anti-HEV IgM. The two anti-HEV IgM-positive donors were HEV RNA-negative. Anti-HEV IgG seroprevalence increases with age (Fig 1). Bivariate analysis (61 variables tested) can be seen in S1 Table. Multivariate analysis identified several characteristics that were independently associated with anti-HEV IgG positivity, Table 1. Increasing age was associated with a positive antibody response (OR>1, p = 0.01). The dietary factors linked to a past or recent HEV infection were the consumption of chicken offal (p = 0.01) and cooked rabbit (p = 0.02). Most striking, the consumption of *fafaru* was associated with a lower rate of positive antibodies (OR = 0.16, p<0.01). More than 99% of donors who ate *fafaru* declared that they ate pork meat, whereas only 90% of those who never ate *fafaru* did so (p<0.01). Among the 300 blood donors, 34 practiced surf or va’a and all were HEV seronegative.

**Table 1:** Factors associated with anti-HEV IgG (Logistic model analysis).

	Regrouped Clinically Selected Variables Associated with Anti-HEV IgG Included in the Logistic Model Analysis					Logistic Model Including Variables Independently Associated with Anti-HEV IgG				
Associated Risk Factor	OR^a^	SE^b^	z	P>|z|	95% CI	OR^a^	SE^b^	z	P>|z|	95% CI
Age	1.04	0.29	1.51	0.13	0.99–1.10	1.05	0.02	2.49	0.01	1.01–1.09
Sex	1.13	0.72	0.19	0.85	0.32–3.91					
Retired person	1.40	1.51	0.32	0.75	0.17–11.54					
Pork sausage	6.27	7.05	1.63	0.10	0.69–56.75					
Beef offal	1.69	1.48	0.60	0.55	0.30–9.41					
Raw chicken meat	1.18	0.82	0.24	0.81	0.30–4.57					
Chicken offal	1.62	1.51	0.52	0.60	0.26–10.08	3.83	2.05	2.51	0.01	1.34–10.96
Cooked rabbit	2.90	1.86	1.60	0.10	0.83–10.17	3.33	1.67	2.39	0.02	1.24–8.91
Goat	0.79	0.63	-0.30	0.76	0.16–3.75					
Pig meat	0.20	0.21	-1.54	0.12	0.03–1.54					
Game	6.84	8.23	1.60	0.11	0.65–72.40					
Fafaru	0.17	0.14	-2.20	0.03	0.04–0.83	0.16	0.11	-2.77	<0.01	0.04–0.58
Raw vegetables	0.85	0.58	-0.23	0.81	0.23–3.21					
Bottled water	0.19	0.21	-1.49	0.14	0.02–1.70					
Tap water	0.55	0.32	-1.02	0.31	0.18–1.73					
Gardening	1.48	0.91	0.63	0.53	0.44–4.96					
Fishing	0.25	0.31	-1.12	0.26	0.02–2.82					
Trip to Europe	0.82	0.58	-0.29	0.77	0.20–3.30					
Trip to Oceania	1.54	0.96	0.70	0.48	0.46–5.20					

^a^ OR: odd ratio

^b^SE: standard error

We explored the interactions in the final model comparing the log likelihood and found no interaction between the variables.

## Discussion

The seroprevalence of anti-HEV IgG among adult Polynesian blood donors in 2014 was low (i.e. 7.7%; 95% CI: [4.9% - 11.3%]) and that of anti-HEV IgM was 0.6% (95% CI: [0.1% - 2.4%]). However, the presence of anti-HEV IgM in 2 Polynesian blood donors, a key marker of recent HEV infection [10], suggest that HEV is circulating in Polynesia.

The seroprevalence of the anti-HEV IgG was lower in French Polynesia than in most European countries but was similar to that of other Oceanian countries. It appears lower than the mean prevalence in French metropolitan blood donors (i.e. 22.4%; 95%CI: 21.6%-23.2%) [7]. In this previous French survey among blood donors, only 3 out of 98 French departments had IgG prevalence <10%. Perhaps the cultural habits of French Polynesians have contributed to the low incidence of HEV in these populations. We also found that older people were significantly more likely to harbour anti-HEV IgG, suggesting a cumulative lifetime exposure to the virus, as previously reported [7, 11].

The link between the consumption of chicken offal and exposure to HEV is in keeping with the results of Wichmann et al., who found that the consumption of any offal, cooked or undercooked was associated with HEV in a German population (p = 0.02) and concluded that it was a major route of HEV transmission [12].

The association between an exposure to hepatitis E and the consumption of cooked rabbit agrees well with the identified causative agents of hepatitis in humans [13, 14]. Only 24.6% of the donors ate rabbit meat—mainly imported, suggesting that eating cooked rabbit is linked to a metropolitan way of life.

Conversely, eating *fafaru* was linked to a lower frequency of anti-HEV IgG. Similarly, all the donors who practice surfing or va’a were HEV seronegative. Eating *fafaru* and surfing or practicing *va’a* can be considered as a proxy of broader cultural habits specific to Polynesians. Some metropolitans may try eating *fafaru*, but the proportion of people eating this Polynesian dish at least once in the year (145/300 donors) corresponds to the proportion of native-born Polynesians. This dietary preference is also related to the Polynesian preference for well-cooked pork, which decreases the risk of infection from pork meat.

We have no indication of what may account for the link between eating *fafaru* and the absence of anti-HEV IgG. While we need ethnicity data in order to formulate an hypothesis, the seroprevalence among Polynesians agrees well with the data from other south Pacific countries [2, 4, 5]. The Polynesian lifestyle and food consumption patterns—especially the very well cooked pork—may be the key [2, 4].

## Conclusions

Our data suggest that HEV exposure appears less common in French Polynesia than in most European countries. The Polynesian lifesyle could help explain this low rate.

## Supporting information

S1 File Questionnaire Part 1. (DOC)

S2 File Questionnaire Part 2. (DOC)

S1 Table Factors associated with anti-HEV IgG (Bivariate analysis). (PDF)
