# Supplementary material for: Hepatitis E prevalence in French Polynesian blood donors
Source: PLoS One. 2018 Dec 7;13(12):e0208934. doi: 10.1371/journal.pone.0208934 (PMC6286134; doi:10.1371/journal.pone.0208934)
Supplement: S1 File — (DOC) [file pone.0208934.s001.doc]

Hepatitis E epidemiological study  


Please not scratch out or write outside of the areas
Please complete the boxes as follows : 


                      
                  
                   Donation number 


Age (years)


Sex                                           M             F


Principal residence                   Appart.           Det. house           Farm            Shelter         Barrack         Other :

Municipality 


Vacation home                         Appart.           Det. house           Farm            Shelter         Barrack         Other :            

Where is your home for weekends or holidays…
Municipality 
/ Island					


Number of members in 
your household                                          including children under the age of 3


Socio-professional category

Farmer
Fisherman
Craftsman –Merchant
Company director
Executive / Liberal Prof. 
Technician / Intermediate Prof.
Employee Worker 
Retired person Student
Other / Inactive	                                   □


1/2
