# Supplementary material for: Hepatitis E prevalence in French Polynesian blood donors
Source: PLoS One. 2018 Dec 7;13(12):e0208934. doi: 10.1371/journal.pone.0208934 (PMC6286134; doi:10.1371/journal.pone.0208934)
Supplement: S2 File — (DOC) [file pone.0208934.s002.doc]

Hepatitis E
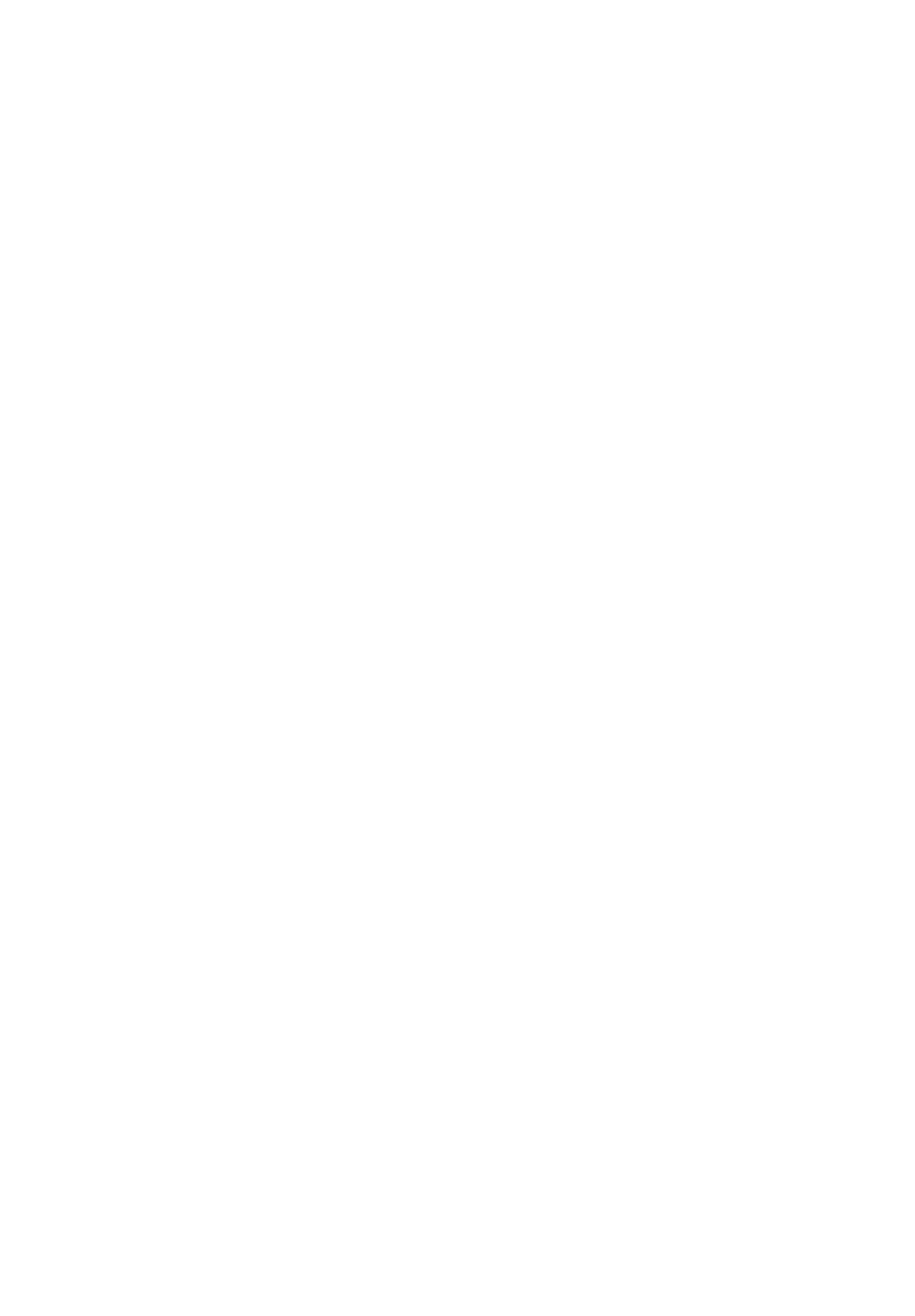
 epidemiological study

*Please not scratch out or write outside of the areas*

*Please complete boxes as follows :*

**Pets □** No

Have you frequent contacts with ? **□** Other:

**□** Cat

**□** Dog

**□** Rodent

**□** Pig

**□** Chicken

**□** Bird

**□** Sheep

**□** Goat

**□** Horse

**□** Cow

**Sewerage**

Does your home have ? **□** Sewer **□** Septic tank **□** Pit to empty **□** Other :

**Dietary habits** What food do you eat ? fresh meat, salted, offal, shellfish and vegetables …

What drinking water do you consume ?

At least 1/week 1/month 1/year never 1/week 1/month 1/year never

**PORK - Puaa**

Meat □ □ □ □

Cooked sausage □ □ □ □

Raw sausage □ □ □ □

Sausage □ □ □ □

Raw ham □ □ □ □

Pâté □ □ □ □

**BEEF or CALF - Puaa toro**

Cooked meat □ □ □ □

Raw meat □ □ □ □

Offal □ □ □ □

**CHICKEN - Moa**

Cooked meat □ □ □ □

Smoked meat □ □ □ □

Offal □ □ □ □

**Fish - Ia**

Cooked □ □ □ □

Raw □ □ □ □

Fafaru □ □ □ □

**SHELLS - SHELLFISHS**

Oysters □ □ □ □

Mussels □ □ □ □

Clams Pahua, Korori □ □ □ □

Troca, Maoa, Pae hoe □ □ □ □

Lobsters Oura miti □ □ □ □

Shrimps Oura pape □ □ □ □

Crabs Kaveu □ □ □ □

Sea Urchins Vana, Rori □ □ □ □

Octopus Fee □ □ □ □

**RABBIT Rapiti**

Cooked meat □ □ □ □

**GAME**

Wild pig □ □ □ □

Goat □ □ □ □

Venison□ □ □ □

Other □ □ □ □

**RAW VEGETABLES**

Unpeeled □ □ □ □

**WATER**

Bottled water □ □ □ □

Tap water □ □ □ □

Source water □ □ □ □

Other □ □ □ □

**HOBBIES** What are your hobbie(s)?

**□** Fishing **□** Hunting **□** Gardening **□** Vegetable gardening **□** Outdoor sport **□** Freshwater bathing **□** Surf **□** Va’a

**TRAVEL W**hich country did/do you already/regularly visit ?

At least Once 1/month 1/year never Once 1/month 1/year never

Europe □ □ □ □

North America □ □ □ □

Central America □ □ □ □

South America □ □ □ □

Oceania □ □ □ □

Asia □ □ □ □

Sub-Saharan Africa □ □ □ □

Maghreb □ □ □ □

**Vaccination Hepatitis A** Have you been vaccinated ? **□** Yes **□** No **□** Don’t know 2/2
