# Supplementary material for: Hepatitis E prevalence in French Polynesian blood donors
Source: PLoS One. 2018 Dec 7;13(12):e0208934. doi: 10.1371/journal.pone.0208934 (PMC6286134; doi:10.1371/journal.pone.0208934)
Supplement: S1 Table — (PDF) [file pone.0208934.s003.pdf]

	IgG negative 277 (92.33)	IgG positive 23 (7.67)	p-value	Total 300 (100.0)
Age (years)			<0.01	
N	277	23		300
Mean	36.19	44.96		36.87
95% CI	34.76-37.63	39.89-50.02		35.47-38.26
Number of member of your household			0.46 ɨ	
N	277	23		300
1/ 1	28 (10.1)	3 (13)		31
2/ 2	48 (17.3)	5 (21.7)		53
3/ 3	52 (18.8)	4 (17.5)		56
4/ 4	69 (24.9)	8 (34.8)		77
5/ 5 or more	80 (28.9)	3 (13)		83
Sex			0.31*	
N	277	23		300
Male	151 (54.5)	10 (43.5)		161
Female	126 (45.5)	13 (46.5)		139
Principal residence			0.35 ɨ	
N	273	23		296
1/ Det. house	60 (22)	7 (30.4)		67
2/ Other	213 (78)	16 (69.6)		229
Number of children under the age of 3			0.30 ɨ	
N	277	23		300
1/ 0	223 (80.5)	22 (95.7)		245
2/ 1	43 (15.5)	1 (4.3)		44
3/ 2 or more	11 (4)	0 (0)		11
Vacation home			0.59 ɨ	
N	277	23		300
1/ Det. house	52 (18.8)	2 (8.7)		54
2/ Other	225 (81.2)	21 (1.3)		246
Socio-professional category			0.72 ɨ	
N	272	23		295
0 / Other/Inactive	39 (14.3)	3 (13)		42
1/ farmer	4 (1.5)	0 (0)		4
2/ craftsman-merchant	9 (3.3)	0 (0)		9
3/ compagny director	8 (2.9)	0 (0)		8
4/ Executive/Liberal prof.	29 (10.7)	4 (17.5)		33
5/ Technician/Intermediate Prof.	21 (7.7)	2 (8.7)		23
6/ employee	105 (38.6)	8 (34.8)		113
7/ worker	5 (1.8)	1 (4.3)		6
8/ retired person	12 (4.4)	3 (13)		15
9/ student	36 (13.2)	2 (8.7)		38

	IgG negative 277 (92.33)	IgG positive 23 (7.67)	p-value	Total 300 (100.0)
Retired person			0.10 ɨ	
N	277	23		300
1/ No	265 (95.7)	20 (87)		285
2/ Yes	12 (4.3)	3 (13)		15
Sewerage				
N	249	23	0.51 ɨ	272
1/ Sewer	49 (19.7)	7 (30.4)		56
2/ Pit to empty	16 (6.4)	0 (0)		16
3/ Septic tank	180 (72.3)	16 (69.6)		196
4/ Other	4 (1.6)	0 (0)		4
Dog			0.40*	
N	271	23		294
0/ No	117 (43.2)	12 (52.2)		129
1/ Yes	154 (56.8)	11 (47.8)		165
Cat			0.31*	
N	277	23		300
0/ No	174 (62.8)	12 (52.2)		186
1/ Yes	103 (37.2)	11 (47.8)		114
Other pet			0.71 ɨ	
N	277	23		300
0/ No	250 (90.3)	22 (95.7)		272
1/ Yes	27 (9.7)	1 (4.3)		28
Pork meat			0.62 ɨ	
N	276	23		299
0/ Never	16 (5.8)	0 (0)		16
1/ At least 1/year	260 (94.2)	23 (100)		283
Pork cooked sausage			0.52 ɨ	
N	275	23		298
0/ Never	35 (12.7)	4 (17.4)		39
1/ At least 1/year	240 (87.3)	19 (82.6)		259
Pork raw sausage			0.57*	
N	275	23		298
0/ Never	172 (62.5)	13 (56.5)		185
1/ At least 1/year	103 (37.5)	10 (43.5)		113
Pork sausage			0.15 ɨ	
N	275	23		298
0/ Never	51 (18.5)	1 (4.3)		52
1/ At least 1/year	224 (81.5)	22 (95.7)		246

	IgG negative	IgG positive	p-value	Total
	277 (92.33)	23 (7.67)		300 (100.0)
Pork raw ham			0.24 ɨ	
N	276	23		299
0/ Never	27 (9.8)	0 (0)		27
1/ At least 1/year	249 (90.2)	23 (100)		272
Pork pâté			0.44*	
N	275	23		298
0/ Never	43 (15.6)	5 (21.7)		48
1/ At least 1/year	232 (84.4)	18 (78.3)		250
Beef cooked meat			1 ɨ	
N	276	23		299
0/ Never	6 (2.2)	0 (0)		6
1/ At least 1/year	270 (97.8)	23 (100)		283
Beef raw meat			0.54*	
N	275	23		298
0/ Never	173 (62.9)	13 (56.5)		186
1/ At least 1/year	102 (37.1)	10 (43.5)		112
Beef offal			0.02*	
N	275	23		298
0/ Never	207 (75.3)	12 (52.2)		219
1/ At least 1/year	68 (24.7)	11 (47.8)		79
Chicken cooked meat			1 ɨ	
N	276	23		299
0/ Never	6 (2.2)	0 (0)		6
1/ At least 1/year	270 (97.8)	23 (100)		293
Chicken smoked meat			0.05*	
N	276	23		299
0/ Never	59 (21.4)	9 (39.1)		68
1/ At least 1/year	217 (78.6)	14 (60.9)		231
Chicken offal			<0.01*	
N	275	23		298
0/ Never	230 (83.6)	13 (56.5)		243
1/ At least 1/year	45 (16.4)	10 (43.5)		55
Rabbit cooked meat			<0.01*	
N	276	23		299
0/ Never	215 (77.9)	10 (43.5)		225
1/ At least 1/year	61 (22.1)	13 (56.5)		74
Goat			0.12*	
N	276	23		299
0/ Never	158 (57.2)	17 (73.9)		175
1/ At least 1/year	118 (42.8)	6 (26.1)		124
Wild pigs			0.06 ɨ	
N	276	23		299
0/ Never	188 (68.1)	20 (87)		208
1/ At least 1/year	88 (31.9)	3 (13)		91

	IgG negative 277 (92.33)	IgG positive 23 (7.67)	p-value	Total 300 (100.0)
Stags			0.67 ɨ	
N	276	23		299
0/ Never	257 (93.1)	21 (91.3)		278
1/ At least 1/year	19 (6.9)	2 (8.7)		21
Game others			0.07 ɨ	
N	276	23		299
0/ Never	266 (96.4)	20 (87)		286
1/ At least 1/year	10 (3.6)	3 (13)		13
Cooked fish			1 ɨ	
N	276	23		299
0/ Never	11 (4)	1 4.3)		12
1/ At least 1/year	265 (96)	22 (93.7)		287
Raw fish			0.29 ɨ	
N	276	23		299
0/ Never	12 (4.3)	2 (8.7)		14
1/ At least 1/year	264 (95.7)	21 (91.3)		285
Fafaru			<0.01 ɨ	
N	276	23		299
0/ Never	134 (48.6)	20 (87)		154
1/ At least 1/year	142 (51.4)	3 (13)		145
Oysters			0.48*	
N	276	23		299
0/ Never	77 (27.9)	8 (34.8)		85
1/ At least 1/year	199 (72.1)	15 (65.2)		214
Mussels			0.29*	
N	276	23		299
0/ Never	58 (21)	7 (30.4)		65
1/ At least 1/year	218 (79)	16 (69.6)		234
Clam shells			0.82*	
N	276	23		299
0/ Never	78 (28.3)	6 (26.1)		84
1/ At least 1/year	198 (71.7)	17 (73.9)		215
Troca			0.42*	
N	276	23		299
0/ Never	132 (47.8)	13 (56.5)		145
1/ At least 1/year	144 (52.2)	10 (43.5)		154
Lobsters			0.62*	
N	276	23		299
0/ Never	73 26.4)	5 (21.7)		78
1/ At least 1/year	203 (73.6)	18 (78.3)		221
Shrimps			1 ɨ	
N	276	23		299
0/ Never	49 (17.8)	4 (17.4)		53
1/ At least 1/year	227 (82.2)	19 (82.6)		246

	IgG negative 277 (92.33)	IgG positive 23 (7.67)	p-value	Total 300 (100.0)
Crabs			0.59*	
N	276	23		299
0/ Never	128 (46.4)	12 (52.2)		140
1/ At least 1/year	148 (53.6)	11 (47.8)		159
Urchins			0.64*	
N	276	23		299
0/ Never	130 (47.1)	12 (52.2)		142
1/ At least 1/year	146 (52.9)	11 (47.8)		157
Octopus			0.89*	
N	276	23		299
0/ Never	184 (66.7)	15 (65.2)		199
1/ At least 1/year	92 (33.3)	8 (34.8)		100
Raw vegetables			0.16*	
N	273	23		296
0/ Never	60 (22)	8 (34.8)		68
1/ At least 1/year	213 (78)	15 (65.2)		228
Bottled water			0.12 ɨ	
N	276	23		299
0/ Never	13 (4.7)	3 (13)		16
1/ At least 1/year	263 (95.3)	20 (87)		283
Tap water			0.01*	
N	275	23		298
0/ Never	67 (24.4)	11 (47.8)		78
1/ At least 1/year	208 (75.6)	12 (52.2)		220
Source water			0.21 ɨ	
N	275	23		298
0/ Never	203 (73.8)	20 (87)		223
1/ At least 1/year	72 (26.2)	3 (13)		75
Other water			1 ɨ	
N	275	23		298
0/ Never	274 (99.6)	23 (100)		297
1/ At least 1/year	1 (0.4)	0 (0)		1
(Vegetable) Gardening			0.15*	
N	277	23		300
0/ No	163 (58.8)	10 (43.5)		173
1/ Yes	114 (41.2)	13 (56.5)		127
Fishing			0.09 ɨ	
N	277	23		300
0/ No	221 (79.8)	22 (95.6)		243
1/ Yes	56 (20.2)	1 (4.4)		57
Freshwater bathing			0.02 ɨ	
N	277	23		300
0/ No	226 (81.6)	23 (100)		288
1/ Yes	12 (18.4)	0 (0)		12

	IgG negative 277 (92.33)	IgG positive 23 (7.67)	p-value	Total 300 (100.0)
Outdoor sport			0.25*	
N	277	23		300
0/ No	178 (64.3)	12 (52.2)		190
1/ Yes	99 (35.7)	11 (47.8)		110
Surf or Va’a			0.09 ɨ	
N	277	23		300
0/ No	243 (87.7)	23 (100)		266
1/Yes	34 (12.8)	0 (0)		34
Travels in Europe			0.12*	
N	276	23		299
0/ Never	131 (47.5)	7 (30.4)		138
1/ At least 1/year	145 (52.5)	16 (69.6)		161
Hunting			0.61 ɨ	
N	275	23		298
0/ No	265 (96.4)	23 (100)		288
1/ Yes	12 (3.6)	0 (0)		12
Travels in North America			0.71*	
N	276	23		299
0/ Never	145 (52.5)	13 (56.5)		158
1/ At least 1/year	131 (47.5)	10 (43.5)		141
Travels in Central America			0.29 ɨ	
N	276	23		299
0/ Never	264 (95.7)	21 (91.3)		285
1/ At least 1/year	12 (4.3)	2 (8.7)		14
Travels in South America			0.29 ɨ	
N	276	23		299
0/ Never	264 (95.7)	21 (91.3)		285
1/ At least 1/year	12 (4.3)	2 (8.7)		14
Travels in Oceania			0.15*	
N	276	23		299
0/ Never	185 (67)	12 (52.2)		197
1/ At least 1/year	91 (33)	11 (47.8)		102
Travels in Asia			0.49 ɨ	
N	276	23		299
0/ Never	246 (89.1)	22 (95.7)		268
1/ At least 1/year	30 (10.9)	1 (4.3)		31
Travels in Sub-Saharan Africa			0.43 ɨ	
N	276	23		299
0/ Never	270 (97.8)	22 (95.7)		292
1/ At least 1/year	6 (2.2)	1 (4.3)		7
Travels in Maghreb			1 ɨ	
N	276	23		299
0/ Never	265 (96)	22 (95.7)		287
1/ At least 1/year	11 (4)	1 (4.3)		12

	IgG negative 277 (92.33)	IgG positive 23 (7.67)	p-value	Total 300 (100.0)
Vaccination Hepatitis A			1 ɨ	
N	110	6		116
0/ No	75 (68.2)	4 (66.7)		79
1/ Yes	35 (31.8)	2 (33.3)		37

ɨ Fisher's exact test

*chi-squared test
